# Exogenous Nitric Oxide Protects Human Embryonic Stem Cell-Derived Cardiomyocytes against Ischemia/Reperfusion Injury

**DOI:** 10.1155/2016/4298945

**Published:** 2016-06-15

**Authors:** János Pálóczi, Zoltán V. Varga, Ágota Apáti, Kornélia Szebényi, Balázs Sarkadi, Rosalinda Madonna, Raffaele De Caterina, Tamás Csont, Thomas Eschenhagen, Péter Ferdinandy, Anikó Görbe

**Affiliations:** ^1^Cardiovascular Research Group, Department of Biochemistry, Faculty of Medicine, University of Szeged, Szeged 6720, Hungary; ^2^Department of Pharmacology and Pharmacotherapy, Semmelweis University, Budapest 1085, Hungary; ^3^Institute of Enzymology, Research Centre for Natural Sciences, Hungarian Academy of Sciences, Budapest 1519, Hungary; ^4^MTA-SE Molecular Biophysics Research Group, Semmelweis University, Institute of Biophysics and Radiation Biology, Budapest 1085, Hungary; ^5^Department of Neuroscience and Imaging, Center of Excellence on Aging, “G. d'Annunzio” University, 66100 Chieti, Italy; ^6^Department of Experimental Pharmacology and Toxicology, University Medical Center Hamburg-Eppendorf, 20246 Hamburg, Germany; ^7^Pharmahungary Group, Szeged 6722, Hungary

## Abstract

*Background and Aims*. Human embryonic stem cell- (hESC-) derived cardiomyocytes are one of the useful screening platforms of potential cardiocytoprotective molecules. However, little is known about the behavior of these cardiomyocytes in simulated ischemia/reperfusion conditions. In this study, we have tested the cytoprotective effect of an NO donor and the brain type natriuretic peptide (BNP) in a screening platform based first on differentiated embryonic bodies (EBs, 6 + 4 days) and then on more differentiated cardiomyocytes (6 + 24 days), both derived from hESCs.* Methods*. Both types of hESC-derived cells were exposed to 150 min simulated ischemia, followed by 120 min reperfusion. Cell viability was assessed by propidium iodide staining. The following treatments were applied during simulated ischemia in differentiated EBs: the NO-donor S-nitroso-N-acetylpenicillamine (SNAP) (10^−7^, 10^−6^, and 10^−5^ M), BNP (10^−9^, 10^−8^, and 10^−7^ M), and the nonspecific NO synthase inhibitor N*ω*-nitro-L-arginine (L-NNA, 10^−5^ M).* Results*. SNAP (10^−6^, 10^−5^ M) significantly attenuated cell death in differentiated EBs. However, simulated ischemia/reperfusion-induced cell death was not affected by BNP or by L-NNA. In separate experiments, SNAP (10^−6^ M) also protected hESC-derived cardiomyocytes.* Conclusions*. We conclude that SNAP, but not BNP, protects differentiated EBs or cardiomyocytes derived from hESCs against simulated ischemia/reperfusion injury. The present screening platform is a useful tool for discovery of cardiocytoprotective molecules and their cellular mechanisms.

## 1. Introduction

Ischemic heart disease is the leading cause of mortality in the Western world; therefore, the development of cardioprotective therapies is currently a main focus of research.* In vitro* cardiac myocyte-based drug-screening platforms are widely used, especially at the early stage of the development of cardioprotective agents. However, these assays are based on cardiomyoblast cell lines or primary neonatal and adult cardiac myocytes [1] and thus have major limitations, including a low proliferation capacity, uncontrolled stress during cell isolation, low throughput, and poor predictability of the assays towards* in vivo* efficacy [2]. Moreover, the relationship between drug responses from animal-derived primary cardiomyocytes and their human counterparts may be significantly different [3]. Human embryonic stem cells (hESCs) are capable of differentiating towards cardiac lineages [4]; therefore, hESCs provide a promising source of cardiomyocytes for* in vitro* drug screening [5, 6]. In addition, hESCs may also provide new tools for regenerative therapies [7–9].

Despite the encouraging results and the enormous potential of the hESC-derived cardiomyocytes, several complications need to be overcome regarding their therapeutic utilization, such as ethical problems, tumor formation, and immunoreactivity. Moreover, it has been demonstrated that the survival of implanted cells is enormously reduced after transplantation [10–12], with these cells undergoing a significant cell death within the first 24 hours [13]. A plausible reason for this effect is the unfavorable microenvironment the grafted cells face when injected into the ischemic host myocardium. Characterization of these cells in an ischemia/reperfusion test system thus would be important, since little is known about the ischemic tolerance of hESC-derived cardiomyocytes.

We have previously shown that the nitric oxide donor S-nitroso-n-acetylpenicillamine (SNAP) and the particulate guanylate cyclase activator B-type natriuretic peptide (BNP) exert a cytoprotective effect against simulated ischemia/reperfusion (SI/R) injury in primary neonatal rat cardiomyocytes [14]. More recently, the cytoprotective effect of SNAP has been shown in mouse embryonic stem cell- (mESC-) derived cardiomyocytes subjected to SI/R treatment [15]. This protection occurs via the activation of protein kinase G (PKG) and stimulation of its downstream signal transduction pathway, which leads to increased cell viability against SI/R injury [14–17]. However, this cytoprotective effect of SNAP and BNP against SI/R injury has not been explored as yet in human cardiomyocytes derived from hESCs.

Therefore, the aim of this present study was to test whether the nitric oxide donor SNAP and the particulate guanylate cyclase activator BNP can protect hESC-derived cardiomyocytes against SI/R injury.

## 2. Methods

### 2.1. Human Embryonic Stem Cell Culture

The CAG promoter driven eGFP expressing human HUES9 stem cell culture (Ethic license: Hungarian Committee of Human Reproduction; 31681-1/2004-1016EHR12534-0/2009-1016EHR; ES2HEART consortium) [18, 19] was dispersed by 0.5 mg/mL collagenase type IV (Gibco, Invitrogen; Carlsbad, CA, USA) dissolved in KnockOut*™* Dulbecco's Modified Eagle Medium (Gibco). Subsequently, cells were maintained in cell suspension culture for 6 days in KnockOut Dulbecco's Modified Eagle Medium (Gibco), supplemented with 20% embryonic stem cell-qualified fetal bovine serum (Gibco), 1% nonessential amino acids, 1% L-glutamine (Gibco), and 0.2% beta-mercaptoethanol (Gibco). To allow clump formation, cell attachment was hampered by using polyhema (5 mg/mL, Sigma; St. Louis, MO, USA) coated surface. After 6 days, the formation of small clumps was observed which are designated as embryonic bodies (EBs).

### 2.2. Differentiation of EBs and Cardiomyocytes Derived from Human Embryonic Stem Cells

Six-day-old EBs were seeded onto gelatin-coated coverslips in 24-well plates. 5–10 EBs were plated into each well. Differentiation of EBs was supported by differentiating media containing Dulbecco's Modified Eagle Medium (Sigma) supplemented with 15% fetal bovine serum (Gibco). EBs were kept under normal conditions (at 37°C, in 95% air and 5% CO_2_ gas mixture) for 4 days prior to SI/R experiments.

In separate experiments, hESC-derived EBs were maintained in differentiating medium for 24 days. At this stage of their differentiation, spontaneous contractions were observed as the sign of the formation of mature cardiac tissues, and these areas were designated as cardiomyocyte-rich region of EBs.

### 2.3. Real-Time PCR Analysis of Differentiated EBs

Cardiac-oriented differentiation of the cells was documented in differentiated EBs by real-time quantitative PCR analysis. Total RNA was isolated from cells using TRIzol*™* reagent (Invitrogen; Carlsbad, CA, USA). Subsequently, cDNA samples were prepared from 1 *μ*g total RNA using the Promega Reverse Transcription System Kit (Promega Corp.; Fitchburg, WI, USA). All these steps were performed according to the manufacturer's instructions. For real-time quantitative PCR, the following predeveloped TaqMan® assays were purchased from Applied Biosystems (Thermo Fisher Scientific; Waltham, MA, USA): octamer-binding transcription factor 4 (OCT4) and the divergent homeodomain protein NANOG as undifferentiated stem cell markers [20–22], BRACHYURY as mesoderm marker [23], the homeobox protein NKX2.5 and the zinc finger transcription factor GATA4 as early markers of cardiac differentiation [24, 25], and activated leukocyte cell adhesion molecule (CD166, ALCAM) as a marker of cardiomyocytes [26]. P0 ribosomal protein was used as endogenous control for quantification. Real-time PCR analyses were carried out using the StepOne*™* Real-Time PCR System (Applied Biosystems), according to the manufacturer's instructions. The fold changes of mRNA in experimental and control cells were determined using the 2^−ΔΔCt^ method. Relative mRNA levels were presented as mean ± SEM of 3 independent experiments.

### 2.4. Immunofluorescence

In order to test the specificity of CAG-driven eGFP expression during cardiac differentiation, immunostaining of cardiac troponin I (cTnI) was performed in 6 + 24-day-old adherent EBs. Samples were fixed with 4% paraformaldehyde in Dulbecco's modified phosphate buffered saline (D-PBS, Sigma) for 15 min at room temperature, followed by three washing steps with D-PBS. To block nonspecific antibody binding, samples were incubated in D-PBS containing 2 mg/mL bovine serum albumin, 1% fish gelatin, 5% goat serum, and 0.1% Triton-X 100 for 1 h at room temperature. The samples were then incubated with primary antibodies (monoclonal mouse anti-cTnI, Sigma) at the dilution of 1 : 500 for 1 h at room temperature. After washing with D-PBS, the cells were incubated with secondary antibodies (Alexa Fluor 568-conjugated goat anti-mouse antibody, Invitrogen) for 1 h at room temperature. Secondary antibodies were diluted in the blocking solution at 1 : 250. 4′,6-Diamidino-2-phenylindole·2HCl (DAPI, Invitrogen) was used for nuclear staining (10 *μ*M, for 10 min in D-PBS). The stained samples were examined by an Olympus fluorescence microscope.

### 2.5. Experimental Groups

**Figure 1:**
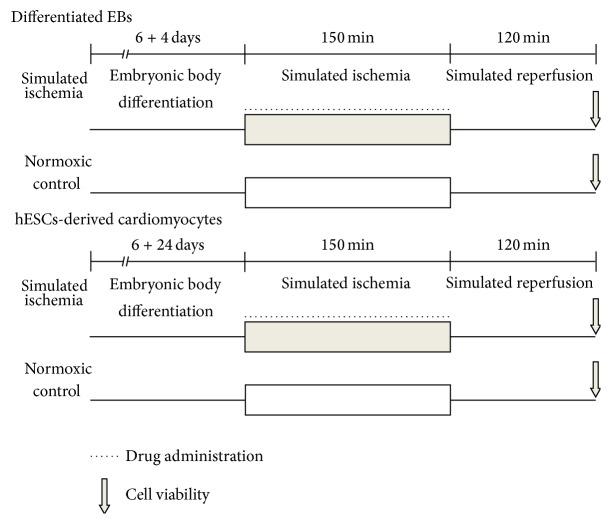
Experimental design of simulated ischemia (SI) and reperfusion (R). hESC-derived EBs (6 + 4 days of differentiation) and differentiated cardiomyocytes (6 + 24 days of differentiation) were exposed to 150 min SI, followed by 120 min R. Cell viability was assessed by propidium iodide staining. The following treatments were applied during SI in differentiated EBs (6 + 4 days of differentiation): the NO-donor S-nitroso-N-acetylpenicillamine (SNAP) (10^−7^, 10^−6^, and 10^−5^ M), BNP (10^−9^, 10^−8^, and 10^−7^ M), and the nonspecific nitric oxide (NO) synthase inhibitor N*ω*-nitro-L-arginine (L-NNA, 10^−5^ M). In case of the hESC-derived cardiomyocytes (6 + 24 days of differentiation), 10^−6^ M SNAP was applied during SI. Viability data were normalized to the cardiac specific CAG-driven eGFP fluorescence.

For cell viability experiments, hESC-derived cells were tested under normoxic condition or were subjected to SI (Figure 1). In normoxic conditions, the differentiating medium was replaced with a normoxic solution (in mM: NaCl 125, KCl 5.4, NaH_2_PO_4_ 1.2, MgCl_2_ 0.5, HEPES 20, glucose 15, taurine 5, CaCl_2_ 1, creatine 2.5, BSA 0.1%, pH 7.4, and 310 mOsm/L) and cells were incubated in a normoxic incubator at 37°C for 2.5 h. Regarding ischemic conditions, the cells were subjected to SI by incubating them in hypoxic solution (in mM: NaCl 119, KCl 5.4, MgSO_4_ 1.3, NaH_2_PO_4_ 1.2, HEPES 5, MgCl_2_ 0.5, CaCl_2_ 0.9, Na-lactate 20, BSA 0.1%, 310 mOsm/L, and pH = 6.4) and exposed to a constant flow of a mixture of 95% N_2_ and 5% CO_2_ for 2.5 hours at 37°C. Cells were subjected to the following treatments during SI: (1) untreated control; (2) SNAP (10^−7^, 10^−6^, and 10^−5^ M) (Sigma); (3) brain type natriuretic peptide-32 (BNP, 10^−9^, 10^−8^, and 10^−7^ M) (American Peptides); (4) NOS inhibitor N-nitro-L-arginine (L-NNA, 10^−5^ M) (Sigma). Concentrations of the compounds were selected according to our previous data [14, 15].

At the second set of experiments, cardiomyocytes derived from hESCs were subjected to the following treatments during SI: (1) untreated control; (2) SNAP (10^−6^ M).

Either normoxic or SI treatments were followed by 2 h reperfusion, when the previously applied solutions were replaced with differentiating medium, and the cells were maintained in a normoxic incubator, gassed with 95% air and 5% CO_2_ at 37°C.

### 2.6. Cell Viability Measurements

At the first set of experiments with differentiated EBs derived from hESCs, after simulated reperfusion, cell viability was assessed by propidium iodide (PI) assay as described previously [15]. Briefly, the growth medium was removed, and the cells were washed with PBS twice and incubated with PI (50 *μ*M, Sigma) for 7 minutes. Each experiment included a digitonin (10^−4^ M, Sigma) treated positive control. Then, PI solution was replaced with fresh PBS and fluorescence intensity of each well was detected by a fluorescent plate reader using 544 ex/610 em filters (FluoStar Optima, BMG Labtech, Thermo Fisher Scientific). PI intensity reflecting cell death was evaluated in the cardiomyocyte-rich region. Since the elevation of eGFP expression is associated with cardiac-oriented differentiation of this hESCs model, the evaluation of cardiomyocyte committed regions was performed manually on each plate by detecting eGFP expression driven by the CAG promoter (Supplemental Figure 1, in Supplementary Material available online at http://dx.doi.org/10.1155/2016/4298945). The cytoprotective effect of different compounds was compared to simulated ischemic control groups, where cell death was designated as 100 percent.

At the second set of experiments, cardiomyocytes derived from hESCs underwent SI/R procedure similar to other groups. Cell viability was assessed by the above-described method.

### 2.7. Statistical Analysis

Results are expressed as mean ± SEM. Unpaired* t*-test and one-way analysis of variance (ANOVA) followed by Fisher's least significant difference (LSD) post hoc tests were used to determine differences in mean values between the experimental groups. Differences were considered significant at *p* < 0.05.

## 3. Results

### 3.1. Cardiac Differentiation of EBs

**Figure 2:**
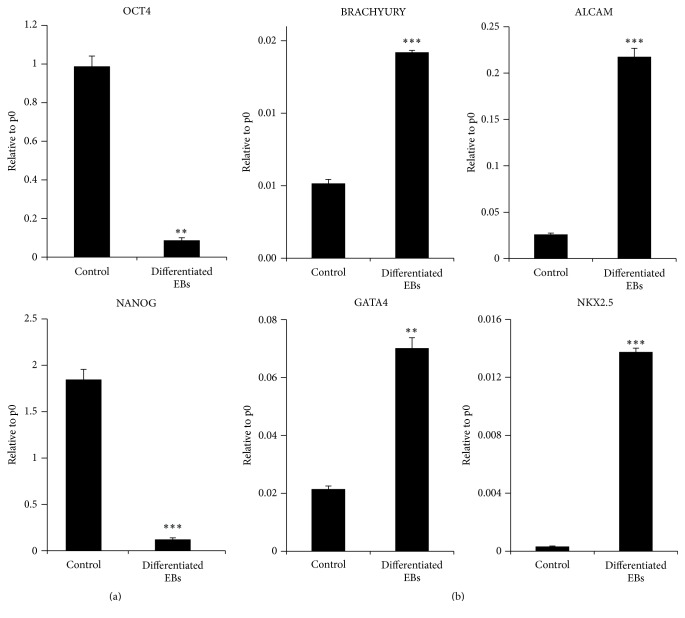
Real-time PCR analysis of differentiated EBs (6 + 4 days of differentiation). OCT4 and NANOG pluripotency markers were downregulated (a), whereas mesodermal (BRACHYURY) and early and later cardiac markers (NKX2.5, GATA4, and ALCAM) were upregulated (b) as compared to the undifferentiated human HUES9 embryonic stem cell line control. Data are expressed as mean ± SEM; ^*∗∗*^ *p* < 0.01, and ^*∗∗∗*^ *p* < 0.001, differentiated EBs versus undifferentiated control; Student* t*-test, *n* = 2.

Real-time quantitative PCR analysis confirmed the cardiac-oriented development of cells in differentiated EBs. Both OCT4 and NANOG pluripotency markers were downregulated in the differentiated EBs as compared to the undifferentiated human HUES9 embryonic stem cell line control (Figure 2). Additionally, the expression of mesodermal (BRACHYURY) and early cardiac markers (NKX2.5 and GATA4) were elevated at this stage of differentiation. Moreover, the cardiac specific ALCAM expression was also upregulated as compared to the control.

**Figure 3:**
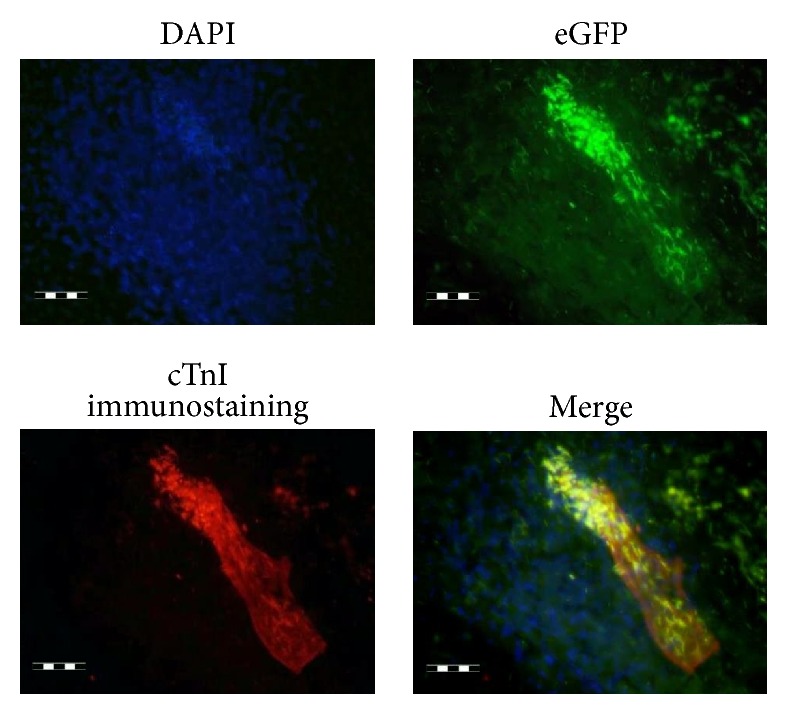
Colocalization of cTnI and enhanced CAG-driven eGFP signals in cardiomyocytes derived from hESCs (6 + 24 days of differentiation). Scale bar: 200 *μ*m.

The specificity of CAG-driven eGFP expression during cardiac differentiation was also documented. In later stage EBs (6 + 24 days), an enhanced eGFP expression was observed in cTnI positive cardiomyocytes derived from hESCs, as confirmed by the colocalization of both signals obtained by immunostaining of cTnI (Figure 3).

### 3.2. Cell Viability after SI/R

The cytoprotective effect of the NO-donor SNAP that activates soluble guanylate cyclase (sGC) was tested in the model of simulated ischemia (SI). Reperfusion-induced cell death was monitored in differentiated EBs as well as in cardiomyocytes derived from hESCs. We found that SI followed by reperfusion caused significantly higher cell death in differentiated EBs or cardiomyocytes than in time-matched controls kept under normoxic conditions (Table 1).

**Figure 4:**
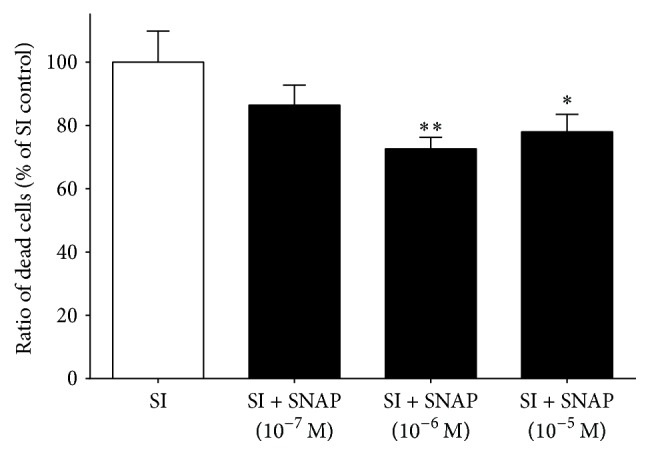
Effect of SNAP on cell viability of differentiated EBs derived from hESCs (6 + 4 days of differentiation). SNAP was applied during SI. Data are expressed as mean ± SEM; ^*∗*^ *p* < 0.05 SNAP treated versus SI control; ^*∗∗*^ *p* < 0.01 SNAP treated versus SI control; one-way ANOVA followed by Fischer LSD post hoc test, *n* = 8 in each group.

In differentiated EBs, cell death was significantly decreased by SNAP in a concentration-dependent manner when applied during the SI period (Figure 4).

**Figure 5:**
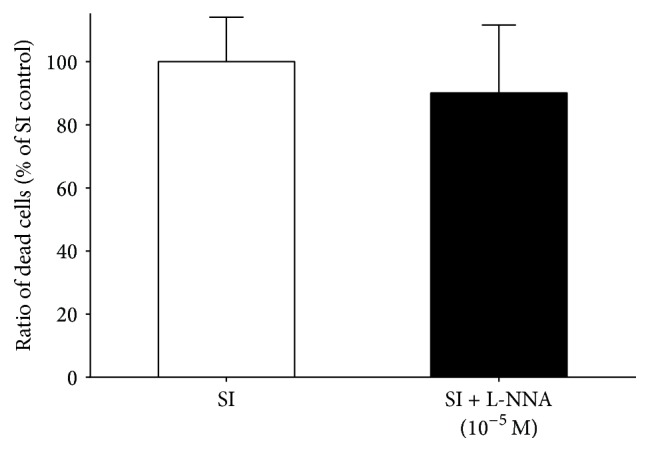
Effect of L-NNA on cell viability of differentiated EBs derived from hESCs (6 + 4 days of differentiation). L-NNA was applied during SI. Data are expressed as mean ± SEM; unpaired* t*-test, *n* = 4 in each group.

**Figure 6:**
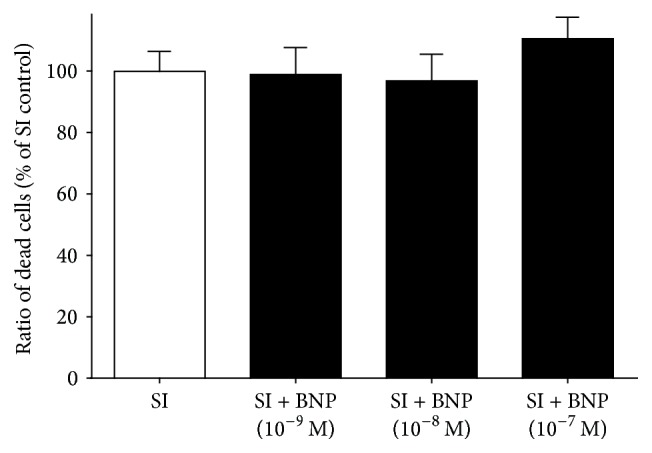
Effect of BNP on cell viability of differentiated EBs derived from hESCs (6 + 4 days of differentiation). BNP was applied during SI. Data are expressed as mean ± SEM, *n* = 8 in each group.

The endogenous NO production was abolished by the administration of the nonselective NOS inhibitor L-NNA at 10^−5^ M concentration. The presence of L-NNA alone did not influence cell death after SI (Figure 5). BNP, an activator of particulate guanylate cyclase tested at 10^−9^, 10^−8^, and 10^−7^ M concentrations, did not influence cell death significantly (Figure 6).

**Figure 7:**
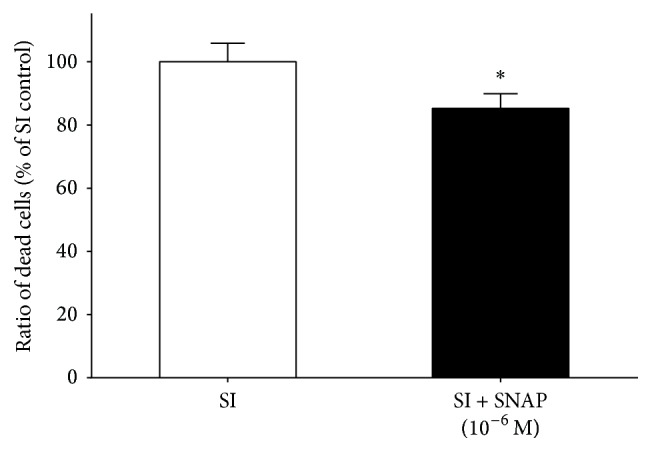
Effect of SNAP on cell viability of hESC-derived cardiomyocytes (6 + 24 days of differentiation). SNAP was applied during SI. Data are expressed as mean ± SEM; ^*∗*^ *p* < 0.05 SNAP treated versus SI control; one-way ANOVA followed by Fischer LSD post hoc test, *n* = 8 in each group.

In order to confirm the cytoprotective effect of SNAP, it was administered to hESC-derived cardiomyocytes. SNAP, as compared to vehicle, attenuated cell death in the hESC-derived cardiomyocytes at 10^−6^ M concentration (Figure 7).

## 4. Discussion

Here, we show for the first time that the NO-donor SNAP is able to provide cardiocytoprotective effect against SI/R-induced cell death in a model of differentiated human EBs as well as in contracting cardiomyocytes derived from hESCs. Moreover, this study is the first demonstration of a hESC-based drug-screening platform that is able to identify cardioprotective compounds against SI/R injury.

Currently used primary cardiomyocyte-based or cardiomyoblast cell line-based drug-screening platforms have many limitations for their utilization. The disadvantages of these assays strongly limit their applicability in preclinical research. Primary neonatal cardiomyocytes are widely used to test cardioprotective drugs; however, results may vary due to culture variability introduced by the isolation procedure or the limited proliferation [27]. Adult cardiomyocytes are suitable to study individual cells, and particularly the pharmacological properties of different ion channels can be examined using this model. However, such cells require special conditions during culturing, especially laminin coated surface, which is indispensable for proper attachment and cell survival [2]. Cell lines are preferable test systems for drug screening, however presenting several limitations. The H9c2 cardiomyoblast line shows different phenotype even from neonatal and adult cardiac myocytes, and additionally spontaneous electric activity and sarcomeric structure cannot be observed in them [28]. Since the translational value of hESC-based platforms for representing human conditions may be substantially higher than the abovementioned cell culture models, here we validated a hESC-derived cardiomyocyte-based drug-screening platform to test cardioprotective agents by using the well-known cardiocytoprotective NO-donor SNAP [14, 15, 29–32].

In our experiments, we found that the NO-donor SNAP increased the viability of differentiated EBs derived from hESCs, subjected to SI/R. This protective effect was dose-dependent, showing the same dose response characteristics as found in neonatal rat cardiomyocytes [14] exposed to SI/R. Our research had shown that NO exerts direct cardiocytoprotection via cGMP-PKG signaling pathway. Moreover, another study on normoxic, neonatal rat cardiac myocyte showed that SNAP caused significant necrosis at 1 mM concentration (no significant changes at 10 and 100 *μ*M), but a bell-shaped effect on apoptosis was observed, that is, significant increase at 100 *μ*M and no effect at 10 *μ*M and 1 mM [33].

Similar results were presented by our group on mouse embryonic stem cell-derived cardiomyocytes, where NO had concentration-dependent direct cytoprotective action and soluble guanylate cyclase, PKG, and KATP channels play a role in the downstream pathway of SNAP-induced cytoprotection [15]. Hsieh et al. showed recently that short pretreatment with NO donor (NaNO_2_) combined with hypoxia protects neonatal cardiac myocyte but not cardiac fibroblast from hypoxic injury, and apoptosis decreased in human ES-derived cardiac myocytes [34]. These results further suggest that NO donors may protect stem cells implanted into ischemic areas of the myocardium. Additionally, it has been shown that NO is able to facilitate ESC differentiation and cardiomyogenesis [35]. To investigate if endogenous NO affects ischemic tolerance of differentiated EBs derived from hESCs, a nonspecific NOS inhibitor, L-NNA, was given during SI. L-NNA did not affect cell viability after SI/R injury of differentiated EBs, indicating that ischemic tolerance of these cells is not altered by endogenous NO. However, local NO concentration largely depends on the ratio of NO and local superoxide production (see, for reviews, Ferdinandy and Schulz, 2003; Andreadou et al., 2015) [36, 37]. Bioavailability of NO also depends on NO synthase (NOS) expression, and previously it was shown that NOS expression has a developmental stage-dependent expression pattern in rat mouse embryos. Mouse EBs treated with NOS inhibitor were prone to less differentiate after embryonic age D-13. Here, we have shown, in human ES-derived cardiac myocytes, that NO has a direct cytoprotective effect in early and more differentiated stage.

Intracellular biosynthesis of cGMP can be catalyzed by both soluble (sGC) and particulate (pGC) guanylate cyclase. The activation of the same intracellular signaling pathway was achieved by administration of BNP (activator of pGC) which is an effective cardioprotective peptide. We have previously shown that exogenously administered BNP reduced cell death after SI/R injury in neonatal rat cardiomyocyte cultures [14] and reduced infarct size at nM concentration range in rat hearts [38]. In agreement with our previous finding in a mouse ESC-derived cardiomyocyte model [15], the exogenously administered BNP was not protective against SI/R-induced cell death in differentiated EBs. The lack of protection may be attributed to the low expression of the NPR-A receptor (specific for BNP) during hESC differentiation [39]. In another study, BNP significantly increased the number of apoptotic neonatal cardiac myocytes subjected to mild hypoxic stimulus (3% O_2_) in a concentration-dependent manner (0.01; 0.1; 1 *μ*mol/L); however, it had no significant effect on the number of necrotic cells [40].

hESC differentiation with suspension EB method results in around 30% cardiac myocyte population [41]; however, the efficacy of cardiac differentiation may vary depending on stem cell line or experimental circumstances [7, 8, 42–44]. Here, in our study, we were able to selectively investigate cardiac myocyte population in the embryonic bodies by using eGFP overexpression, which enhances the specificity of screening platform.

In summary, here, we demonstrate for the first time that SNAP, but not BNP, protects differentiated human EBs or cardiomyocytes against SI/R injury. Our findings also suggest that hESC-derived differentiated EBs containing early cardiac committed cells may serve as a screening platform for the discovery of cardiocytoprotective molecules; additionally, the present platform is suitable for testing the cardiac myocyte population of the EBs.

## Supplementary Material

Supplemental Figure 1: Representative images of viability assay of differentiated EBs (6+4 days of differentiation). Panel A shows cardiac oriented differentiation of EBs as indicated by higher eGFP fluorescence. Panel B represents propidium iodine (PI) fluorescence following SI/R injury. High PI fluorescence (shown in yellow or in red) reflects severe cell death. Evaluation of the viability of cardiomyocyte committed regions was performed manually on each plate by detecting eGFP expression.

## Figures and Tables

**Table 1a:** (a) The effect of SI/R on cell death of differentiated EBs derived from hESCs (6 + 4 days of differentiation)

Group	Mean RFU ± SEM	*p* value
Normoxia	1141 ± 69.83	*p* = 0.0019
SI	3624 ± 516.2	(unpaired *t*-test)

**Table 1b:** (b) The effect of SI/R on cell death of hESC-derived cardiomyocytes (6 + 24 days of differentiation)

Group	Mean RFU ± SEM	*p* value
Normoxia	65817 ± 10272	*p* = 0.0027
SI	137045 ± 17555	(unpaired *t*-test)
