# Supplementary figures and images for: Exogenous Nitric Oxide Protects Human Embryonic Stem Cell-Derived Cardiomyocytes against Ischemia/Reperfusion Injury
Source: Oxid Med Cell Longev. 2016 Jun 15;2016:4298945. doi: 10.1155/2016/4298945 (PMC4925993; doi:10.1155/2016/4298945)

## Slide 1
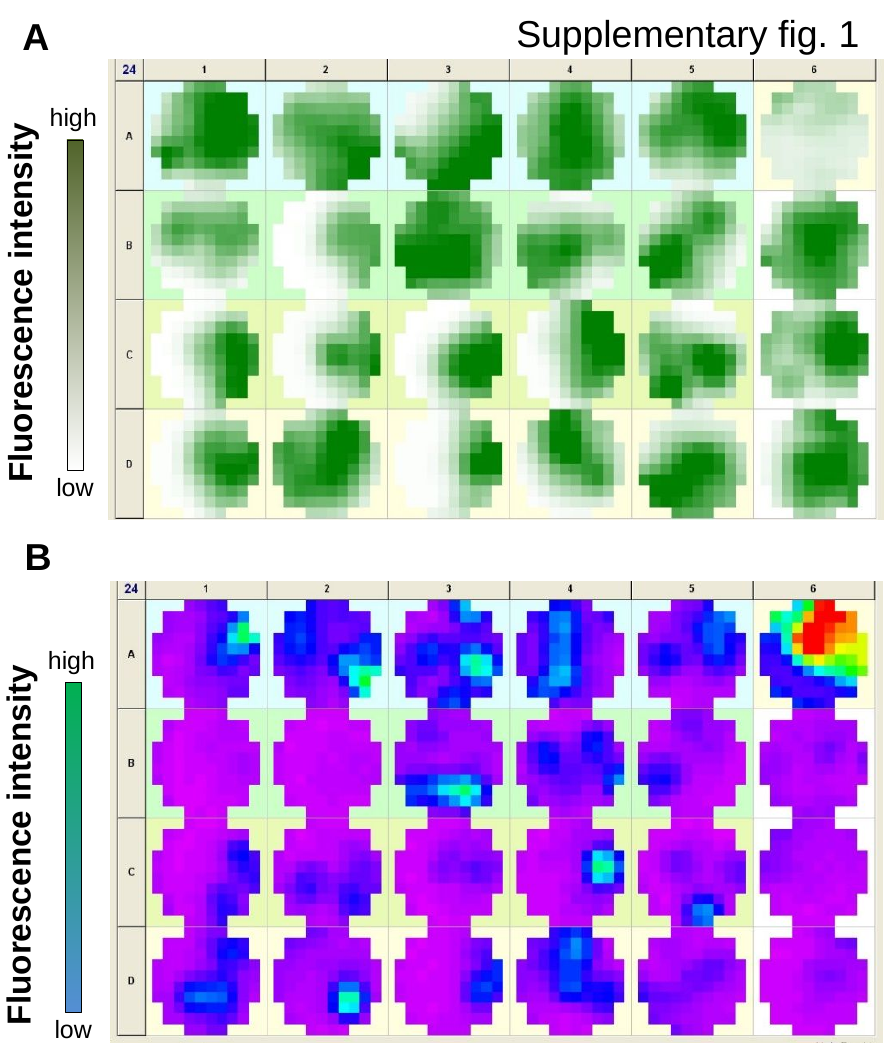

Supplementary fig. 1
A
high
Fluorescence intensity
low
B
high
Fluorescence intensity
low

Supplement: Supplementary file 1 — Supplemental Figure 1: Representative images of viability assay of differentiated EBs (6+4 days of differentiation). Panel A shows cardiac oriented differentiation of EBs as indicated by higher eGFP fluorescence. Panel B represents propidium iodine (PI) fluorescence following SI/R injury. High PI fluorescence (shown in yellow or in red) reflects severe cell death. Evaluation of the viability of cardiomyocyte committed regions was performed manually on each plate by detecting eGFP expression. [file 4298945.f1.pptx]
